# Teleophthalmology Service: Organization, Management, Actual Current Applications, and Future Prospects

**DOI:** 10.1155/2021/8876957

**Published:** 2021-06-03

**Authors:** Raffaele Nuzzi, Davide Bovone, Fabio Maradei, Paolo Caselgrandi, Alessandro Rossi

**Affiliations:** Institute of Ophthalmology, Department of Surgical Sciences, University of Turin, Turin, Italy

## Abstract

Teleophthalmology (TO) consists of the clinical and therapeutic approach to the patient (e-Health) using informatic and telecommunication systems. Already widespread worldwide, it aims to improve patient care, expand the healthcare offer, and access to medical care by reducing overall costs. Despite the organizational, legal, and management difficulties, the substantial economic investments necessary for the start-up of equipped structures and efficient territorial services are amply rewarded by economic results and optimal services for professionals and patients. This review specifically analyses the current scenario of teleophthalmology, the points for and against its application in different sociodemographic realities, and in particular, the current and future fields of use.

## 1. Introduction

Telemedicine (TM) is the set of medical and informatic techniques that allow you to perform a medical examination and provide health services to a patient remotely. In the context of clinical diagnostics, it is possible for a doctor to make the diagnosis on a patient who is not physically in the same place as the doctor, through the remote transmission of data produced by diagnostic instruments. Second medical opinion is one of the most common applications in the field of telemedicine: it consists in providing a remote clinical referral supported by acquired data sent to a remote doctor who analyses and reports them.

There are already many fields of application of TM: an example is teleradiology, in which an instrumental examination is performed (RX, CT, MR) by qualified personnel, and the images are acquired and sent electronically to a remote radiologist who visualizes them, analyses them, and reports the exam, then sending the report to the Specialist Center that performed the exam or directly to the patient. The same method is applied in telecardiology, where an electrocardiogram (ECG) is performed and transmitted remotely; other examples may be teledermatology, teleneurology, or even telerehabilitation, which consists in the provision of rehabilitation services through telecommunication networks and the internet [1].

The primary objective of telemedicine, possible thanks to the enormous technological development of the last 30-40 years, was to allow adequate assistance in the most remote geographical areas or in difficult situations (e.g., oil drilling on offshore platforms, artic, or space expeditions). Subsequently, with the spread of more effective data compression techniques and faster networks, it was also possible to send bulky data via the fixed network, such as images of a computerized tomography (CT).

In Italy, one of the first telemedicine applications consisted in the experimental transmission of remote ECG, which began in 1976, using normal telephone lines. Later, in the eighties, SIP (“Società Italiana per le telecomunicazioni”, Italian telecommunications company) launched a real “cardiotelephone.” Since then, research institutes, universities, scientific societies, the National Research Council (CNR) ,and the Ministry of Health, working on various projects, have led to the activation of a master's degree in telemedicine in some universities (for example, in Pisa), to the organization of important conferences on the topic and to the exponential growth of the available services.

In this regard, the great advances in technology combined with the growing interest in TM have meant that smartphone applications, now essential tools for life, became an integral part of e-Health, more precisely, of m-Health (mobile-Health).

These clinical-medical applications allow the user to manage his own health through his mobile phone, educating him especially on what attitudes and behaviors follow for the prevention of various diseases: equally important are those useful applications programmed for monitoring the progress of chronic diseases such as arterial hypertension (through the possibility of having a blood pressure diary, judgments on the trend for those who are learning about their condition, information material on hypertension, programs to reduce blood pressure following an adequate lifestyle) or diabetes (possibility of having a diary with glycemic data, with attached notes on meals taken and teaching materials useful for improving one's health).

In recent years, a start-up founded by young Italians invented “BOCA health,” an application for smartphones, useful to the management of the human body fluids in patients suffering from heart failure, by improving the patients' journey from hospital to home. The components are a portable multifrequency composition analyzer, wearable and reusable electrodes, and an app that records the data and sends it to the doctor remotely who, after analyzing them, will optimize remote therapy, thus avoiding the patient to go to hospital or to his medical practitioner, with a considerable saving of time and economic resources for both the patient and public health. This innovation therefore allows to filter the clinical situations that require hospitalization from those that do not require it, thus avoiding the patient journeys to the hospital and the possible nosocomial infections related to them and allowing him to manage the situation comfortably from his home thanks to the optimization of therapy by your doctor remotely.

This review aims to present the principles of teleophthalmology, the application models underlying its organization. The fields of current application and the legal aspects that control clinical practice and protect patient data will then be presented.

## 2. Principles of Teleophthalmology

Teleophthalmology (TO) consists of the clinical and therapeutic approach to the patient (e-Health) using informatic and telecommunication systems. Already widespread worldwide, it aims to improve patient care, expand the healthcare offer, and access to medical care by reducing overall costs. International Studies reveal that 93% of eye diseases can be detected thanks to automated tests and that in about 80% of cases, it is possible to put into practice effective prevention through early screening. Despite this, more than 285 million people (5% of the population) worldwide suffer from undiagnosed eye diseases [2].

The development of TO therefore has a twofold objective: the first, at an international level, aims to bring medical skills and the possibility of diagnosis and treatment in countries with limited economic opportunities, with extremely large territories and with low availability of dedicated healthcare personnel. Numerous projects are currently active in some reality such as the Chinese, Indian, Brazilian, and South African ones for the screening and remote management of pathologies such as diabetic retinopathy, age-related macular degeneration, and retinopathy of the premature baby, with good results regarding early diagnosis, management, and follow-up. The second objective, at local level, is to facilitate access to diagnostic procedures in countries that already have an organized health system but are increasingly finding it difficult to meet the needs of the aging population. Examples have been such as Great Britain and Sweden, where strategies are already being implemented using TO in daily medical practices [3, 4].

Despite the increasing number of data supporting the effectiveness of the TO, numerous problems represent challenges, such as the nonhomogeneous distribution of telecommunication infrastructures, the purchase costs of the necessary equipment, the control of accuracy of machinery and diagnostic appropriateness, lack of guidelines and trained personnel, and legal issues relating to privacy and security. There are also many doubts about the adequacy of a medicine that seems to deplete the doctor-patient relationship, from the point of view of both the specialist and the patient [1].

### 2.1. Smart Applications and Working Model

An example of applied teleophthalmology is undoubtedly the one devised by the Greek doctor Iordanis Chatziandgelidis based on a remotely controlled slit lamp (SL). Greece has more than 200 islands, in which hospitals are mostly primary care centers and do not count ophthalmological services: this means that if a resident of those islands needs a visit or an eye exam, it must face a long expensive journey (10-12 hours), by ship, which is not guaranteed every day and varies according to sea conditions, only to reach the specialist center located in the nearest city. This difficulty in traveling made these journeys justified only in cases of extreme urgency, instead leaving out less complicated but no less serious situations, which if they had been taken in time would have been resolvable [5]. Doctor I.C. developed a remotely controllable SL. Simply using a second-hand SL, a movement controller, and a smartphone, he developed the first fully self-financed prototype and presented it at the ESCRS Winter Meeting in Ljubljana in 2014. His goal was to bring this project to the attention of the Greek government for the inclusion of the protocol in the NHS, but he received just some compliments or little more. Fortunately, a local company decided to invest in his idea, making it possible to produce and market his project. In February 2018, ITACA was born, with clear reference to the homeland of the mythical Ulysses [5].

### 2.2. How It Works

Before starting the eye examination, the doctor and the patient meet through an app like Facetime or Skype for a first cognitive meeting. According to Dr. I.C., the doctor-patient relationship remains a fundamental part of the medical examination, greatly improving the patient's compliance [5]. During the interview, the doctor collects the patient's medical and ophthalmological history, as well as the symptoms and disorders. Trained personnel takes care of detecting visual acuity, intraocular pressure, and performing the Amsler test. Subsequently, using the 4G network and a smartphone fixed on the SL, the specialist, assisted by the health staff on site, can start the examination by checking the SL remotely through a joystick hooked to his cell phone. The ophthalmologist is thus able to illuminate the patient's eye and to see the image on the screen, to increase or decrease the intensity of the illumination, to change the magnification and to move the SL in the longitudinal, lateral, and angular. This allows the specialist to highlight the problem “live” and give an immediate response to the patient, with the possibility of indicating the appropriate therapy immediately and/or prescribing other tests to complete the diagnostic/therapeutic path to solve the problem, obviating therefore to the problem of distance, travel and the cost related to it, and to the possible complications deriving from neglecting the pathology [5].

### 2.3. Italian Scenario

As for the Italian panorama, an example of applied TO is IGOR® (Internet Group Ophthalmology Report), created in 2013 by the innovative start-up from Novara. IGOR® is based on the collaboration between selected diagnostic centrs (IGOR® Point-IP) and certified ophthalmologists (IGOR® Specialist–IS) [6]. The patient registers for free by creating a personal account on the web or via mobile app and geolocates the nearest IP. Without any reservations, trained staff in these centers submits the patient to the required diagnostic examination. The images are immediately uploaded to the servers of the Operations Center, and the IS network is automatically activated, guaranteeing the issuance of the report on the patient's personal account within 72 hours. IS accesses exams remotely and evaluates them using simple standard masks. If the exam is doubtful or the images are of poor quality, the exam can be repeated, free of charge for the patient. In case of needs, it is possible to choose the IS and possibly request a second opinion. The patient is always suggested to leave feedback on the quality of the service offered. As a control system, every month, a Scientific Committee of specialists reevaluates a percentage of sample exams. The costs to be paid by the patient are lower than the average costs of the regional health tickets [6]. Among the tests that can be performed at the IP there are optical coherence tomography (OCT), computerized visual field (CVF), digital and autofluorescence retinography (AF), corneal topography, and slit lamp digital imaging (Pic2Aask®). The system, by encrypting the patient's sensitive data, is designed to record the flow of exams by recording for each single transaction data such as type of exam, image quality, patient age, pathology and severity, ongoing therapies, IP, IS, times of reporting and delivery, approval rating, and many others. The degree of satisfaction with the service provided is then assessed by analyzing a questionnaire consisting of 20 questions completed by the patients [6].

What emerges from these projects that are already active in the Italian and International field is that TM in general, and TO in particular, allows to bring an advantage from the health point of view both to those poor or particularly large countries, making treatment accessible to those with more difficulties, and to those countries already with a good NHS but where the widespread presence on the territory allows to lighten public health by first level exams by sending them to operators trained in located centers [6]. In this way, accessibility to the service is improved, and a waiting list reduction is guaranteed; the patient can take advantage of an easy-to-find and use service quickly and with considerable economic advantage. Savings are made not only in terms of money but also in terms of time and travel. In addition, the creation of a digital archive is an innovation that simplifies and makes more efficient of the management of medical documents for healthcare professionals and for the patient himself. The TO represents the adaptation to modern times of an ancient discipline, which today has assumed a peculiar connotation with a high technological content, without running the risk of diminishing the profession of the doctor as such. In fact, it must always be kept in mind that this system allows the doctor to obtain instrumental examination reports quickly and easily, which, however, alone are not sufficient to make a diagnosis of pathology nor to deny it. The supervision of the ophthalmologist is in fact necessary for the interpretation and integration of the data collected according to the personal evaluation of the individual patient [6].

## 3. Health Organization for Telemedicine in Ophthalmology

The advent of telemedicine as a new diagnostic device support in well under way and fully structured hospital ward entails many complex organizational changes that may take up both economically and logistically the medical unit [7]. The same issues arise when a new telemedicine diagnostic hotspot is set up. In the latter case, the need to install new pieces of machinery, new operating rooms, and the training of new personnel makes the investment needed higher. Such investments should be followed by several advantages such as economic savings and provision of higher quality services [8]. This is the equivalent exchange always demanded to a new technique that wishes to achieve among other goals progress.

The organizational issues that must be taken into consideration involve all the healthcare personnel that usually cooperate during a face-to-face medical examination. The assumption that the advent of telemedicine in ophthalmology might reduce the need of healthcare and technical personnel is erroneous. What really changes is the skills that all the active operators must develop. Thus, to ensure economic savings and provision of higher quality services, the logistics of the ward, the organization of both the healthcare personnel, and the patients must be revised.

In order to fully rely on ophthalmic telemedicine, it is important to distinguish those patients that need a face-to-face examination; thus, it is of vital importance to periodically verify all the telemedicine protocols. As well as all the other diagnostic techniques, all the parameters evaluated must be confronted with the ones obtained with the current gold standard technique. To do so, both sensitivity and specificity must be taken into consideration. Ribeiro et al. [4] focused their attention on ocular emergencies: they used teleophthalmology to rapidly screen patients that lived in remote and underserved areas in Brazil. The study found that teleophthalmology had 92.85% sensitivity, 81.94% specificity, 85% accuracy, 66.66% PPV (positive predictive value), and 96.72% NPV (negative predictive values) in detecting ocular emergencies.

### 3.1. Territorial Organization Models

Many medical centers are working to adapt the preexisting structure for telemedicine. Some diseases such as cataract [9], glaucoma [10], diabetic retinopathy [11], and other retinal affections [11] lend themselves well to telediagnosis. What is common about these diseases is the possibility to solely evaluate the images following well defined protocols, without the need to catty out a face-to-face examination.

A medical center that has been carrying out face-to-face examination already has all the tools and pieces of machinery needed for telediagnosis. The introduction of teleophthalmology only requires the shift of office visits into virtual referrals. The internal logistics is composed of first phase in which the healthcare operators explain to the patient how the examination will be carried out; subsequently, some nurses who have been well trained [11] on how to use the pieces of machinery (such as OCT [12, 13] and digital cameras [13]) collect the sensitive data and start capturing of the images. At the end of the examination, the patients leave the center. At first, all the data is collected in the center's server; it is then sent to the central server in which all the data is stored. The latter may be consulted by medical staff from every web portal to carry out the diagnosis. The transmission of the data through the 4G system and/or broadband allows the medical staff to immediately evaluate the imaged and makes it possible to carry out the diagnosis 24/7 [13]. Both the doctors and the patient may consult all the medical documents at any time; they can work remotely and consult the previous reports without the need of the hard copy. These advantages are not confined to ophthalmology: all the other specialists that examine the patients may connect to the central database and consult the reports.

The majority of the works that have analyzed this new system [11] report that a face-to-face examination followed by a virtual report is the best solution in terms of efficacy and selection of patients according to the severity of the illness. All those patients that must undergo an instrumental procedure (ex. laser) or surgery (ex. cataract) or that must change therapy or are not compliant to the current one (ex. glaucoma) may be contacted by the doctor in order to carry out a face-to-face examination [13]. On the average, the number of patients that requires a second examination in a specialized center is 30% [14]. Nonetheless, some authors report a significantly lower percentage (2%) [14] thus implying that the internal logistics make a great difference in providing a high-quality service, especially for diseases such as cataract. All the patients that do not need a face-to-face examination are redirected to scheduled virtual follow-up.

The system with the best outcomes [14] in terms of efficacy of the hotspot center, dedicated to the acquisition of prediagnostic images, recommends the following model:

- First, it is of vital importance to obtain the inform consent. After that, a detailed medical history is taken. Only then can visual acuity and intraocular pressures be recorded; by using a great variety of fundus cameras, many digital photographs are taken
- If a specific ocular disease is identified some additional photographs of the affected area are captured
- Once the examination is over, all the images are automatically named and stored. In some cases, it is the technician who has to store in the correct place the images by using a drag and drop software
- All the images are encrypted, and the file is uploaded onto a password-protected secure server (http://www.teleophthalmology.com/).
- When the physician is ready to examine the pictures, the file is uncompressed and extracted into subfiles. The images are processed so as to ensure a stereoscopic presentation. The image files are processed with ImageJ software, then sorted by image field and organized for web-based stereoscopic presentation in slide show format for the physician grader to review
- Stereo viewing is achieved by viewing images on a dual monitor workstation. The grader allows to maximize visualization by zooming, adjusting brightness, etc.
- Once all the images have been examined, a PDF is automatically created; it can then be faxed or e-mailed as a password-protected web link
- The ophthalmologist that graded the images will be then compensated via a fee-for-service arrangement with the provincial health care provider

This simple yet practical organization allows a complete distinction between the diagnostic hotspots and the specialized centers. By doing so, the latter can focus on the more difficult cases and on the patients that need a surgical intervention [9]. The aim of distinguishing the two diagnostic phases is to centralize the pieces of information and the most difficult cases in specialized centers only when it is absolutely necessary, carrying out follow-up examination in a more practical and economical way both for the patient and the national health systems [9]. The main source of economy derives from the lower amount of hours that must be paid to the medical staff for carrying out the same number of examinations (what must be noticed is that all the waiting lists [11] for new examinations, follow-up and check-ups will be shorter). In addition to this, the cost savings will derive from the repetitiveness of the organization of the hotspots, the lower number of accesses to complex structures, a lower number of complications, and a shorter average time spent [14, 15] at the hospital which leads to lower exposure to the related risk factors. This economic mode has already shown its cost effectiveness [7]: all the resources saved can be reallocated to enhance the specialized centers and invested on the training of the personnel. In order for the system to be fully efficient, there must be many numbers to support the hotspots. The higher the number of accesses to the centers, the higher the savings [15]. In addition to all that has been reported is of vital importance for the sustainability of the system that the hotspots are accurate in selecting the patients recommended for virtual follow-up. In literature, it is accepted a percentage up to approximately 40% of patients recommended for clinical reexamination. It is reported that the most frequent causes of requirement of face-to-face examination are poor image quality (13%), fundus changes (17%), and images that suggested eye fundus alterations but are not clear enough for a save diagnosis (10%). The latter, when correctly identified, represents the quality parameter of the system [14].

The adaptative effort requests to the structures are not limited to the economical investment needed for the pieces of machinery and the training of the personnel [15]: it should also focus on the protection of the data collected. The development of specific internal protocols drafted following the supranational guidelines in force is of vital importance to ensure the data confidentiality to the users. Furthermore, the structures should work on defining a protocol that includes a program of upgrading of the operative system and of the hardware components, thus ensuring an efficient standardization along with quality levels higher than those reported in the centers that carry out only examinations face to face [16].

The side effect often reported derives from the “assembly line effect.” This may undermine the professionalism of the care provided, lowering excessively the perceived medicalization level. For this reason, the structures must consider a specific training for doctors, nurses, and technicians [17], and the patients should be fully informed on the assistance level they will receive that in fact ensures in a technological way the experience of highly specialized centers without the need to reach it in person. An active network of professionals in the territory (nurses and doctors) already exists in a lot of countries.

In terms of territorial applications, it will be of common interest to activate a communication network for pediatric ophthalmology. In Italy, as in many countries, the pediatric network is very well established and distributed throughout the territory thanks to free specialist clinics that rely on major facilities for instrumental diagnostics. Decentralizing the instrumental screening phases on the territory would make it possible to replicate the active model for adult teleophthalmology, with extreme ease, even for the pediatric population.

### 3.2. Patient Protocols

Many authors have focused their attention on evaluating the pros and cons of teleophthalmology. One of the most innovative and useful features that it offers is the possibility to ensure a high leveled diagnostic service even in the most rural and inaccessible areas. Not only does the doctor diagnose the images received, but he is at the disposal if the patient needs a face-to-face examination. The great amount of clinical data collected requires the development of a reliable database. It might be useful to collect and store the data in an easy way so that all the patients will be able to get access to their images and medical report [18]. The immediate availability of the data allows the doctors that will carry out other examinations on the patient to consult the previous reports and read the patient's history without having seen him [7]. Lee et al. [17] report that 75% of patients considers teleophthalmology an opportunity to share the data with more than one doctor and to be fully aware of the disease thanks to the immediate feedback of the examination [7]. Furthermore, many patients, especially the old and the frail, see in telemedicine an opportunity to be examined properly by going to the nearest center, avoiding the risks involved with going to a major clinical center (infections, falls…). Moreover, the shortening of the waiting lists is perceived by the patients as a better and more efficient service.

Indeed, there are some cons that must be pointed out. Some of these are the perception that the patient has of the data management, the depersonalization of the service, the replacement of a confidential of the doctor-patient relationship with the simple compilation of a report, and the collecting of clinical data, most of which are not followed by a face-to-face examination which might only have the aim of reassuring a pluripathological patient, especially in those patients affected by a degenerative disease which might lead to blindness.

Some of the issues that have been raised can be easily solved. The data transparency and the share of the regulation of the process of personal data must follow the European legislation that has been in force for years. Its development has been the subject to review in the last few years in all developed countries. The patient must have the opportunity to resort to older forms of examination and not be forced to be exposed to the risk of losing some data or the risk of hackerization which, however minimal, is still present.

When focusing on the ethical aspects of this new concept of medical examination, it cannot be forgotten that there might be an erroneous interpretation of the medical reports. The patient that receives an immediate response and has access to all of his data might live his disease in a counterproductive way. This aspect is linked to the novelty effect, which will gradually unravel as soon as teleexaminations become part of the patient's daily life. The risk of depersonalization must be supplied by a correct training of the nurses and technicians that capture the images and collect the patient's data. In fact, they will be in charge of the concept of “care” that will be fully distinct from the concept to “cure” which is left to those who will analyze the data collected. These two sides of a coin must not seem split at the patient's eye. The latter should perceive a full availability of receiving a face-to-face examination in order to fulfill the wish of confidentiality acquired with the examiner. This aspect must be considered especially for the elderly that might be more reluctant and unwilling to accustom to modern technology, but at the same time domiciled in those rural areas in which telemedicine is more useful. The start-up of a public or private structure of teleophthalmology must be accompanied by a training [17] not only of the staff but also of the patients to whom it is addressed so that everybody is aware of the seriousness of the report [19] as well as the efficacy of the therapy.

The satisfaction surveys that have been analyzed reveal a good satisfaction rating; although, some of them have a small sample size [14].

### 3.3. Organization and Management of the Staff

The development of delediagnosis centers involves doctors, nurses, and technicians. In the hotspot organizational model, the healthcare personnel is committed to a work of great professionalism. In fact, following a careful and detailed training session, it will be them who must run the whole examination and the human contact. If there is not the suspect of a potentially evolutive disease and/or the need of intervention, the doctor's role in the virtual referral phase will be only decisional. This organizational model empowers the nurses with a fundamental role in ensuring the quality of the service [16, 20]; on the other hand, it allows the doctors to concentrate on the decisional aspects of the examination. Some of the money saved by the advent of telemedicine may be reinvested by the second and third level centers for research purposes and for the development of highly specialized sections. Thanks to telemedicine that its highly qualified competence may reach effectively wide rural areas that were previously deficient in resources and personnel and did not receive the same quality services as other areas or towns.

The data sharing system also allows other specialists to immediately consult the reports uploaded and cooperate in the management of the patient. Furthermore, through telemedicine, distant working different specialists may discuss and come up with the best conclusion for the patient. This liaison between different specialists ensures the attainment of a higher degree of diagnostic accuracy, thus leading to a better service provided by both the hotspots and the second- and third-degree centers. The cornerstone of such a dynamic service is the sharing of compatible communication systems.

As regards to the medical staff, the diversity of the working interface allows a precise scheduling of the working day, with more predictable timelines, diminishing of the work-related stress and a variation of the repetitiveness of the examinations that usually mines the working activities.

On the other hand, the medical staff may report a depersonalization of their role, an erroneous perception of the seriousness of the problem and loss of contact with the patient. Therefore, it is fundamental to have put into effect an appropriate training for the healthcare personnel and to have a solid organization to support it.

Another problem that must be pointed doubt is the risk of the constant availability. The shared data will allow the specialist to access the data at any time of the day; Although, this makes telediagnosis fast and efficient, especially in emergency cases, but there are chances that the doctor becomes on-call 24/7 [9]. Nevertheless, the use of specific access protocols can easily get around the problem.

In summary, by comparing teleophthalmology with the current screening programs, there are three main benefits:

- Not necessary referrals will be minimized
- After having undergone surgery or any other form of intervention, the patient can be monitored at a distance without the need for travelling and avoiding further risks exposure
- Through a teleophthalmology examination, a face-to-face visit or even an intervention might be planned at the discretion of the specialist

Telemedicine poses a challenge to all the structures that decide to put their resources in this technological progress. It requires a complex management system that must take into consideration moral and ethical along with practical and logistic aspects. The certainty of a financial return [9] and the possibility to ensure a high-quality service [20, 21] are enough to encourage the structures to develop teleophthalmology programs. The biggest challenge that the structures must face in the years ahead is to maintain and strengthen the safety of the data stored [18] and convinces all the software and hardware producers to ensure the highest compatibility with the systems currently in use [22].

## 4. Current Applications of Teleophthalmology

The progressive increase of chronic ocular pathologies, associated with an increase in life expectancy and an increase in the average age of the population, means that more and more people are at risk of visual impairment and blindness on the part of pathologies such as age-related macular degeneration (AMD), diabetic retinopathy, and glaucoma. The relative lack of ophthalmologists and the serious economic consequences resulting from the increase in these chronic diseases has allowed teleophthalmology to become established as a screening and evaluation method.

Several studies have confirmed that the introduction of teleophthalmology allows to identify and therefore to treat some chronic eye diseases earlier, for example, through the use of home monitoring devices [18]. Teleophthalmology also makes it possible to extend care to a greater portion of the population, also reaching areas far from large urban centers [23]. These aspects potentially translate into a decrease in costs for the health system and a reduction in the economic and social weight related to the decrease and loss of vision [24].

The meta-analysis conducted by Kawaguchi et al. concludes that teleophthalmology as a screening method for the main chronic eye diseases, compared to the traditional eye examination, does not present statistically significant differences in terms of diagnostic accuracy, but presents a greater participation by the population [25]. A recent review states that 16-48% of traditional eye examinations could be avoided if a teleophthalmological triage occurred earlier [26].

Currently, the main applications of teleophthalmology consist in the screening and management of the most frequent chronic eye diseases, such as diabetic retinopathy, AMD, and glaucoma.

### 4.1. Diabetic Retinopathy

Diabetic retinopathy (DR) is the most common microvascular complication of diabetes and is a major cause of vision loss in adults aged 20 to 74 years globally [27]. It is estimated that the number of people with DR worldwide will amount to over 190 million by 20301. Some clinical trials have shown that early diagnosis and early treatment of DR can reduce the risk of serious vision impairment by more than 50%10; other studies report that early laser treatments and intravitreal injections based on anti-vascular endothelial growth factor can reduce the risk by more than 90% [28].

A big problem is patients' lack of disease awareness: a recent survey revealed that 73% of patients with diabetic retinopathy in the United States are unaware of their condition [29]. The goal of teleophthalmology in DR screening is therefore to increase the number of diagnoses and monitor any disease progressions in these patients15. Some countries such as the United Kingdom and France have already started successful screening programs for DR using telemedicine [30].

In the literature in most studies on the screening of DR through teleophthalmology, “store and forward” methods are used [31]. First a trained technician or nurse collects images of the fundus from the patient using a mydriatic or non-mydriatic retinal chamber, then archives them on a device supplied to the patient or sends them to a server, along with other additional information such as the visual acuity, eye tone and anamnestic data. Remotely the general practitioner (GP) or an ophthalmologist analyses the images. It is often the GP who first views the images and refers only the most complex cases to the ophthalmologist; a clinical study reports that when this occurs, only 30% of the initial cases are referred back to the ophthalmologist [31].

The screening effectiveness of DR can be significantly increased with the introduction of OCT [32]. The possibility of adding OCT to the images of the ocular fundus allows early detection of any macular oedema and immediate start of intravitreal treatment.

Various studies and a recent meta-analysis have shown that teleophthalmology is a convenient screening method for DR with high diagnostic precision [33].

### 4.2. Age-Related Macular Degeneration

Age-related macular degeneration (AMD) is the leading cause of blindness in individuals 50 yr and older in developed countries [34]. Again, teleophthalmology represents a great opportunity for screening and early diagnosis, given that AMD has an asymptomatic onset and most patients with AMD are unaware of their condition [35].

A recent prospective clinical trial conducted by Li et al. compared teleophthalmological screening with a traditional retinal visit in identifying the change in AMD from the dry to the neovascular form [36]. The study did not highlight differences between the two groups, and this suggests that teleophthalmology may be a useful tool for the early detection of neovascular AMD [36].

Also, in this case, some studies have investigated the cost-benefit ratio of a possible introduction of OCT, concluding that with the latter, there is a reduction of 34% of patients who need to be seen by an ophthalmologist, showing an overall reduction of costs [37].

Teleophthalmology in AMD screening also uses home monitoring devices, such as the Amsler grid or the ForeseeHome device (Notal Vision Ltd., Tel Aviv, Israel). The latter is a device introduced by the AREDS2-HOME study [38] that allows you to perform a macular visual field test as home monitoring and that transmits data to be analyzed remotely. The study has shown that the use of this device has made it possible to recognize early forms of neovascular AMD in high-risk patients [38].

### 4.3. Glaucoma

Glaucoma is another major cause of irreversible blindness, and it is estimated that more than 60 million people are affected worldwide [39]. Early diagnosis and the start of treatment are essential to avoid vision loss, which typically occurs asymptomatically.

**Figure 1:**
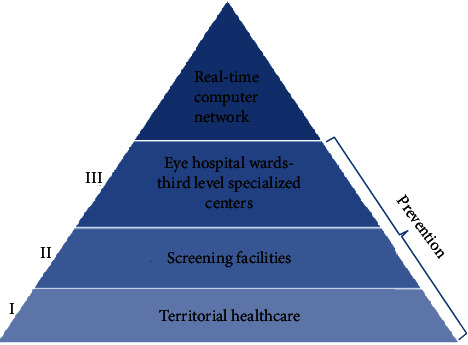
Glaucoma prevention: the primary prevention should be mainly a competence of the territorial healthcare, the secondary prevention should be carried out by screening facilities, and the third prevention should be operated by the eye hospital wards and the third level specialized centers. The essential link that unites and connects all these identities shall be the creation of a real-time computer network.

Prevention at all levels is therefore essential in this area. Primary and secondary prevention is implemented at the local level, through territorial healthcare and screening programs, while tertiary prevention occurs in the ophthalmology departments and in the specialized centers for glaucoma, once the diagnosis has been made. It is equally important to establish a communication network that can correctly insert and guide patients within this path, first of prevention and then of treatment (Figure 1) [40].

**Figure 2:**
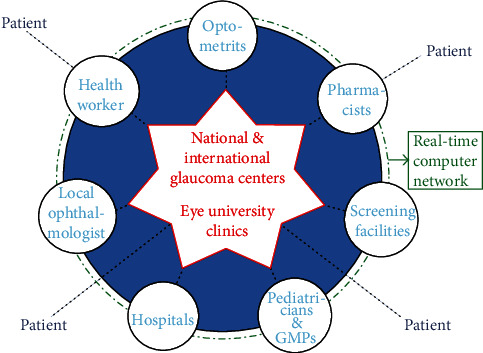
The “hub and spoke” model of glaucoma management: the hubs correspond to optometrists, pharmacists, pediatricians, general medical practitioners (GMPs), the local ophthalmologist, health workers, screening facilities, and hospitals, while the spoke is represented by the national and international glaucoma centers and the Eye University Clinics.

The management of glaucoma should be carried out according to the “Hub and Spoke” theory. This theory provides that the patient, once visited by the local health facilities, is directed to the reference centers for the specific pathology while maintaining a constant and effective communication network at all levels. The communication network should involve several professionals, both health and nonhealth (Figure 2).

In this case, teleophthalmology can provide for the collection of images of the optic disc through portable cameras, OCT of the optic nerve fibers, and visual field tests. Home monitoring devices for intraocular pressure (IOP) have also been introduced, which allow the data collected to be stored or sent directly to the clinical reference center. In 2016, contact lenses were marketed with an integrated sensor that allows you to record the IOP continuously over the course of 24 hours. They are soft silicone lenses that record the circumferential changes of the corneoscleral surface and on the basis of these derive the IOP values. However, these technologies are still under development and significant differences between the various devices still remain [41].

A recent meta-analysis has shown that teleglaucoma screening has a higher specificity and lower sensitivity in the diagnosis of glaucoma compared to a traditional eye examination. Teleophthalmology allows a reduction in the duration of the visit, reduces the patient's travel time, and is overall more convenient [36].

### 4.4. Other Applications

Another eye disease that can benefit from telemedicine is retinopathy of prematurity (ROP), in which it is possible to take high resolution images of the fundus via mobile devices. Beyond ROP, a clinical study proposed extending the screening with photographs of the fundus to all infants and not only those at risk of ROP, showing that almost 5% of infants have ocular changes that require eye medical attention [41].

Eye emergencies are another example of the application of teleophthalmology. Some emergency departments are equipped with a slit lamp and a chamber for the ocular fundus, and other departments are simply equipped with a mobile phone with dedicated lenses. Ophthalmologists remotely use the patient's medical history and images made by emergency department staff to triage patients and discriminate against those who need a specialist visit. A review has shown that safe and accurate triage decisions are made with this system [42].

The use of teleophthalmology also allows to reduce direct contact between the doctor and the patient, and this is of decisive importance for reducing the transmission of infectious diseases. During the COVID-19 pandemic, the use of teleophthalmology has significantly increased and has also found application in the field of uveitis. Patients with uveitis are generally more fragile patients as in 30-45% of cases, and they have an underlying autoimmune, neoplastic, or infectious condition from which ocular inflammation derives. Moreover, many of these patients require the use of immunosuppressive drugs and therefore can benefit in particular in limiting interpersonal contacts since they have an increased risk of contracting infections. However, there are still several significant technological limitations that prevent the standard visit for uveitis from being completely replaced.

## 5. Legal and Medical Aspects of Teleophthalmology

The applications of telemedicine in ophthalmology are undoubtedly promising and will be increasingly expanded. Parallel to the evolution of applications and programs, it was necessary to deepen the subject also from an ethical and medical legal point of view, given the need for the acquisition and management of personal data, medical records, and images. To date, the legal substrate of activities related to telemedicine is based on rules and agreements in force in the European context, first of all, the General Data Protection Regulation (GDPR).

Teleophthalmology also has important consequences from an ethical point of view, given the different approach in the relationship between doctor and patient. It is necessary to ensure the fiduciary aspect between the patient and the doctor, avoiding the risk of “withdraw” of these two figures. The service of teleophthalmology needs an organizational structure that certainly does not involve only the doctor but also other figures such as technicians, health facilities, and insurance. In this enlarged reality, patient protection must be ensured, as well as the proper management of sensitive personal data [43].

From a medical-legal point of view, the regulations to which teleophthalmology must refer are the same as those of telemedicine. The collection and use of sensitive patient data through electronic instruments for teleophthalmology are regulated in Italy by the provisions of Legislative Decree no. 196/2003 and, at European level, by the GDPR. The Legislative Decree 196/2003 indicates methods and solutions necessary to protect confidentiality, integrity, and availability of data. This legislative decree sets out the minimum security features necessary for the processing of sensitive data collected by electronic means and indicates the mandatory provisions necessary for the use of these services in accordance with the law [43].

The management of data, the collection of informed consent, and the possibility of transmitting information to different structures remain fundamental aspects for the correct use of telemedical services.

To preserve the confidentiality and personal identity of patients, in addition to the abovementioned topics, three fundamental aspects are also needed [44]:

- The informed consent
- The information on treatments
- Rights of the patient to their personal data

Informed consent is an authorization given by the patient after being informed about the risks and expectations of the clinician to receive any healthcare treatment. This document is also necessary for the collection of data and the use of teleophthalmology technologies. In this specific case, it must clearly indicate who is the person or organization that collects, processes, and transmits the data, the intermediaries that will have access to the information, the expectations, and the reasons that make it necessary to use the teleophthalmology and actions aimed at the protection of sensitive personal data. In accordance with Legislative Decree 219/2017, informed consent is mandatory for any medical act with respect for dignity and individual freedom, which is why also the case of teleophthalmology requires its collection and subscription by the interested patient. In accordance with the General Data Protection Regulation (GDPR), following the signing of informed consent, the patient can no longer refuse data analysis for purposes of diagnosis and treatment. Despite this lack of possibility of refusal, the processing of personal data requires anonymity, and the creation of a digital document is accessible only to those who have the authorization and for the uses permitted by current legislation [45].

In clinical practice, the information on treatments mainly concerns the risk/benefit ratio of a medical act, the possible complications, and the description, of the necessary procedures. In the case of teleophthalmology, on the other hand, privacy needs focus mainly on data management, processing, for diagnosis and treatment uses, and the possibility of access to individuals not involved in the various teleophthalmology projects. In response above all to patients' fears about the disclosure and free access of their data, the European Union and the various States individually intervened to codify procedures that allow compliance with current regulations [46]. Already in the act of consent, but especially during the information to interested patients, must be very clear how data collection is made, from which subjects can be viewed and processed and what security measures are used to protect the information. With regard to the information on treatments, it would be preferable to use texts as uniform as possible, especially at national level but also at European level, given the significant technological progression that allows communication between health care providers in countries even far away [47].

Clear, simplified, and organized procedures are desirable for the understanding of patients' rights to their personal data. All information that suggests the physical and mental health of the patient, including genetic information and photographs, shall be considered as such [6]. All these data, as established by the judgment of the Italian Supreme Court no. 30981 of 2017 [48], are to be considered as sensitive data and as such need to be treated with procedures such as encrypting, which allow anonymity and not traceability to the patient's identity. In accordance with European legislation (GDPR), the information collected can only be used for diagnosis, treatment, and supervision by national health services and for research purposes. All other purposes, such as preventive medicine and occupational medicine, do not meet the eligibility criteria for data collection according to current legislation.

Approved in July 2016, the NIS (Network and Information Security) Directive is a milestone for the start of standardization between different states regarding the control of the transmission and acquisition of sensitive data. The NIS directive imposes at European level the adoption of common security measures between the different European states and, for each state, the creation of CSIRT (Computer Security Incident Response Team) that supervises all critical networks. The goal is certainly to reduce the risk in cases of network violations especially as a result of the exponential increase in the spread of connected devices [48, 49].

From a medical-legal point of view, the regulations to which teleophthalmology must refer are the same as those of conventional medicine. In fact, the technologies that allow teleophthalmology are comparable to a medical act; according to the European Definition of Medical Act (EDMA) that is to be considered a medical act in any activity aimed at promoting health, preventing disease, and diagnosing and treating individuals, groups, or communities, according to ethical standards.

The regulatory requirements of teleophthalmology concern above all the protection, the management of sensitive data, and the safety of networks, a very dear topic to patients and often feared by clinicians. To date, various laws regulate this complex reality, and data security is the cornerstone for further development of future applications of ophthalmology [50].

## 6. Conclusions

In recent years, telemedicine and teleophthalmology have demonstrated their ability to improve the service without worsening the diagnostic accuracy of all the professionals involved. The constitution of this valid alternative combined with a careful organization can give way to a new model of health organization for the eye examination. From an economic and social point of view, the medium and long-term advantages of the new healthcare organization models are now indisputable. The high number of pathologies that can be managed with teleophthalmology techniques and the high prevalence of these pathologies allow for maximum optimization of the structures involved. The legal aspects involved obviously require the utmost attention to guarantee the patient the highest standard of treatment together with the maximum protection of his personal data.

## References

[1] Wilson L. S., Maeder A. J. (2015). Recent directions in telemedicine: Review of trends in research and practice. *Healthcare Informatics Research*.

[2] Bittner A. K., Yoshinaga P. D., Wykstra S. L., Li T. (2020). Telerehabilitation for people with low vision. *Cochrane Database of Systematic Reviews*.

[3] Flodgren G., Rachas A., Farmer A. J., Inzitari M., Shepperd S. (2015). Interactive Telemedicine: Effects on Professional Practice and Health Care Outcomes. *Cochrane Database of Systematic Reviews*.

[4] Ribeiro A. G., Rodrigues R. A. M., Guerreiro A. M., Regatieri C. V. S. (2014). A teleophthalmology system for the diagnosis of ocular urgency in remote areas of Brazil. *Arquivos brasileiros de oftalmologia*.

[5] Gaspari L. (2019). When technology and ophthalmology reduce distances. *Eyesee*.

[6] Fanton G., Bruschini E., Galli L. (2016). Teleophthalmology made in Italy: IGOR, the protagonist. *Tomorrow Ophthalmology*.

[7] Kanjee R., Dookeran R. I., Mathen M. K., Stockl F. A., Leicht R. (2017). Six-year prevalence and incidence of diabetic retinopathy and cost-effectiveness of teleophthalmology in Manitoba. *Canadian Journal of Ophthalmology*.

[8] Li Z., Wu C., Olayiwola J. N., Hilaire D. S., Huang J. J. (2012). Telemedicine-based digital retinal imaging vs standard ophthalmologic evaluation for the assessment of diabetic retinopathy. *Connecticut medicine*.

[9] Pathak S., Kumar B. (2017). Wireless teleophthalmology: a novel, low-cost, flexible network architecture and its performance evaluation for remote eye care solutions. *Telemedicine and e-Health*.

[10] Hark L. A., Myers J. S., Rahmatnejad K. (2018). Philadelphia telemedicine glaucoma detection and follow-up study: analysis of unreadable fundus images. *Journal of glaucoma*.

[11] Kern C., Kortuem K., Hamilton R. (2019). Clinical Outcomes of a Hospital-Based Teleophthalmology Service: What Happens to Patients in a Virtual Clinic?. *Ophthalmology Retina*.

[12] Kiernan D. F., Mieler W. F., Hariprasad S. M. (2010). Spectral-Domain Optical Coherence Tomography: A Comparison of Modern High- Resolution Retinal Imaging Systems. *American journal of ophthalmology*.

[13] Medical Advisory Secretariat (2009). Optical coherence tomography for age-related macular degeneration and diabetic macular edema: an evidence-based analysis. *Ontario Health Technology Assessment Series*.

[14] Sreelatha O. K., Ramesh S. V. S. (2016). Teleophthalmology: Improving Patient Outcomes?. *Clinical Ophthalmology*.

[15] Trikha S., MacGregor C., Jeffery M., Kirwan J. (2012). The Portsmouth-based glaucoma refinement scheme: a role for virtual clinics in the future?. *Eye*.

[16] Clarke J., Puertas R., Kotecha A., Foster P. J., Barton K. (2017). Virtual clinics in glaucoma care: Face-to-face versus remote decision-making. *British Journal of Ophthalmology*.

[17] Lin C. T., Albertson G. A., Schilling L. M. (2001). Is patients’ perception of time spent with the physician a determinant of ambulatory patient satisfaction?. *Archives of internal medicine*.

[18] Holekamp N. M. (2018). Moving from clinic to home: what the future holds for ophthalmic telemedicine. *American journal of ophthalmology*.

[19] Court J. H. A. M. (2015). Virtual glaucoma clinics: patient acceptance and quality of patient education compared to standard clinics. *Clinical Ophthalmology*.

[20] Leese G. P., Morris A. D., Swaminathan K. (2005). Implementation of national diabetes retinal screening programme is associated with a lower proportion of patients referred to ophthalmology. *Diabetic Medicine*.

[21] Bowman R. J., Kennedy C., Kirwan J. F., Sze P., Murdoch I. E. (2003). Reliability of telemedicine for diagnosing and managing eye problems in accident and emergency departments. *Eye*.

[22] De Fauw J., Ledsam J. R., Romera-Paredes B. (2018). Clinically applicable deep learning for diagnosis and referral in retinal disease. *Nature Medicine*.

[23] Chin E. K., Ventura B. V., See K. Y., Seibles J., Park S. S. (2014). Nonmydriatic fundus photography for teleophthalmology diabetic retinopathy screening in Rural and Urban Clinics. *Telemedicine and e-Health*.

[24] Jani P. D., Forbes L., Choudhury A., Preisser J. S., Viera A. J., Garg S. (2017). Evaluation of diabetic retinal screening and factors for ophthalmology referral in a telemedicine network. *JAMA ophthalmology*.

[25] Kawaguchi A., Sharafeldin N., Sundaram A. (2018). Teleophthalmology for age-related macular degeneration and diabetic retinopathy screening: a systematic review and meta-analysis. *Telemedicine and e-Health*.

[26] Caffery L. (2019). Models of care in tele-ophthalmology: a scoping review. *Journal of Telemedicine and Telecare*.

[27] Ting D. S., Cheung G. C., Wong T. Y. (2016). Diabetic retinopathy: global prevalence, major risk factors, screening practices and public health challenges: a review. *Clinical & Experimental Ophthalmology*.

[28] Wells J. A., Glassman A. R., Ayala A. R. (2016). Aflibercept, bevacizumab, or Ranibizumab for diabetic macular edema two-year results from a comparative effectiveness randomized. *Ophthalmology*.

[29] Singer D. E., Nathan D. M., Fogel H. A., Schachat A. P. (2017). Screening for diabetic Retinopathy. *North Carolina Medical Journal*.

[30] Chabouis A., Berdugo M., Meas T. (2009). Les benefices d'Ophdiat^®^, un reseau de telemedecine pour le depistage de la retinopathie diabetique : analyse retrospective dans cinq centres hospitaliers. *Diabetes & Metabolism*.

[31] Villa S. R., Álvarez C. A., Del Valle R. D. (2016). Five-year experience of teleophthalmology for diabetic retinopathy screening in a rural population. *Archivos de la Sociedad Española de Oftalmología (English Edition)*.

[32] Manjunath V., Papastavrou V., Steel D. H. W. (2015). Wide-field imaging and OCT _vs_ clinical evaluation of patients referred from diabetic retinopathy screening. *Eye*.

[33] Shi L., Wu H., Dong J., Jiang K., Lu X., Shi J. (2015). Telemedicine for detecting diabetic retinopathy: a systematic review and meta-analysis. *The British Journal of Ophthalmology*.

[34] Wong W. L., Su X., Li X. (2014). Global prevalence of age-related macular degeneration and disease burden projection for 2020 and 2040: A systematic review and meta-analysis. *The Lancet Global Health*.

[35] Gibson D. M. (2012). Diabetic retinopathy and age-related macular degeneration in the U.S. *American journal of preventive medicine*.

[36] Rathi S., Tsui E., Mehta N., Zahid S., Schuman J. S. (2017). The current state of Teleophthalmology in the United States. *Ophthalmology*.

[37] Kelly S. P., Wallwork I., Haider D., Qureshi K. (2011). Teleophthalmology with optical coherence tomography imaging in community optometry. Evaluation of a quality improvement for macular patients. *Clinical Ophthalmology*.

[38] Chew E. Y., Clemons T. E., Bressler S. B. (2014). Randomized trial of a home monitoring system for early detection of choroidal neovascularization home monitoring of the eye (HOME) study. *Ophthalmology*.

[39] Ittoop S. M., JR S. H., Seibold L. K., Mansouri K., Kahook M. Y. (2016). Systematic review of current devices for 24-h intraocular pressure monitoring. *Advances in Therapy*.

[40] Nuzzi R., Marolo P., Nuzzi A. (2020). The hub-and-spoke management of glaucoma. *Frontiers in Neuroscience*.

[41] Vinekar A., Govindaraj I., Jayadev C. (2015). Universal ocular screening of 1021 term infants using wide-field digital imaging in a single public hospital in India - a pilot study. *Acta Ophthalmologica*.

[42] Kulshrestha M., Lewis D., Williams C., Axford A. (2010). A pilot trial of teleophthalmology services in North Wales. *Journal of Telemedicine and Telecare*.

[43] Decreto legislativo 30 giugno 2003, n. 196 riguardo Codice in material di protezione dei dati personali

[44] Gioia G., Salducci M. (2019). Medical and legal aspects of telemedicine in ophthalmology. *Romanian journal of ophthalmology*.

[45] EHealth Stakeholder Group Widespread deployment of telemedicines services in Europe (2014). *Report of the eHealth Stakeholder Group on implementing the digital agenda for Europe. Key action 13/2 “Telemedicine.”*.

[46] Morse A. R. (2014). Telemedicine in ophthalmology: Promise and pitfalls. *Ophthalmology*.

[47] COCIR Telemedicine toolkit (2010). *European Coordination Committee of he Radiological, Electromedical and Health Care IT Industry*.

[48] Cassazione civile, SS.UU., sentenza del 27/12/2017 n° 30981.

[49] Directive NIS 2016/1148, Official journal, 09-06-2018, General Series n. 132.

[50] NIS directive (EU) (2016). 2016/1148 of the European parliament and of the council of 6 July 2016 containing measures for a high common level of security of networks and information systems in the Union. *EN Official Journal of the European Union*.

